# Tissue- and Condition-Specific Isoforms of Mammalian Cytochrome *c* Oxidase Subunits: From Function to Human Disease

**DOI:** 10.1155/2017/1534056

**Published:** 2017-05-16

**Authors:** Christopher A. Sinkler, Hasini Kalpage, Joseph Shay, Icksoo Lee, Moh H. Malek, Lawrence I. Grossman, Maik Hüttemann

**Affiliations:** ^1^Center for Molecular Medicine and Genetics, Wayne State University School of Medicine, Detroit, MI 48201, USA; ^2^College of Medicine, Dankook University, Cheonan-si, Chungcheongnam-do 31116, Republic of Korea; ^3^Department of Health Care Sciences, Eugene Applebaum College of Pharmacy and Health Sciences, Wayne State University, Detroit, MI 48201, USA

## Abstract

Cytochrome *c* oxidase (COX) is the terminal enzyme of the electron transport chain and catalyzes the transfer of electrons from cytochrome *c* to oxygen. COX consists of 14 subunits, three and eleven encoded, respectively, by the mitochondrial and nuclear DNA. Tissue- and condition-specific isoforms have only been reported for COX but not for the other oxidative phosphorylation complexes, suggesting a fundamental requirement to fine-tune and regulate the essentially irreversible reaction catalyzed by COX. This article briefly discusses the assembly of COX in mammals and then reviews the functions of the six nuclear-encoded COX subunits that are expressed as isoforms in specialized tissues including those of the liver, heart and skeletal muscle, lung, and testes: COX IV-1, COX IV-2, NDUFA4, NDUFA4L2, COX VIaL, COX VIaH, COX VIb-1, COX VIb-2, COX VIIaH, COX VIIaL, COX VIIaR, COX VIIIH/L, and COX VIII-3. We propose a model in which the isoforms mediate the interconnected regulation of COX by (1) adjusting basal enzyme activity to mitochondrial capacity of a given tissue; (2) allosteric regulation to adjust energy production to need; (3) altering proton pumping efficiency under certain conditions, contributing to thermogenesis; (4) providing a platform for tissue-specific signaling; (5) stabilizing the COX dimer; and (6) modulating supercomplex formation.

## 1. Introduction

Mammalian mitochondria are remarkable cellular organelles, possessing a unique, conserved genome distinct from the nuclear genome, as well as providing the means for energy generation theorized as a principle requirement for the advent of multicellular organisms [1]. The evolution of the electron transport chain (ETC) together with ATP synthase—a series of large multisubunit protein complexes responsible for oxidative phosphorylation (OxPhos)—was pivotal in this development, increasing the amount of adenine triphosphate (ATP) generated from the oxidation of glucose by ~15-fold compared to fermentative processes [2]. The ETC consists of three proton pumps, NADH dehydrogenase (complex I), *bc*_1_-complex (complex III), and cytochrome *c* oxidase (COX; complex IV). In addition, the ETC contains succinate dehydrogenase (complex II), which feeds electrons from succinate into the ETC but does not pump protons, and the small electron carriers cytochrome *c* and ubiquinone.

In addition to their essential function in aerobic energy metabolism, mitochondria have been found to have vital functions in apoptosis [3–7], aging [8–10], and numerous diseases ranging from cancer [11] to diseases involving ischemia/reperfusion injury [12] to inflammation [13, 14] and sepsis [15–17]. Within the context of the cell itself, mitochondria perform multiple functions beyond the scope of oxidative phosphorylation, including calcium modulation and sequestration [18–20] as well as production of reactive oxygen species (ROS). ROS have been implicated in numerous pathways as an essential signal [14, 21–23] and further as an important regulator of mitochondrial proteins, including COX biogenesis and assembly [24]. Cellular production of ROS can be directly modulated by uncoupling the electron transport function from OxPhos or by attenuating mitochondrial electron flow through the addition of respiratory inhibitors [25, 26]. It is important to note that despite directly interacting with dioxygen, COX itself is not known to generate ROS—this function is specifically linked to the NADH dehydrogenase and *bc*_1_ complexes of the ETC [27–29]. Given the inherently dangerous nature of ROS and their ability to modify nonspecific targets, defense mechanisms exist to attenuate their destructive potential. Superoxide dismutase and catalase exist to degrade ROS products into less reactive forms, while glutathione, thioredoxin, and other thiols exist to act as buffering agents [30–32]. Together, these systems represent a vital component of a balanced system that must be tightly regulated.

Mitochondria themselves are thought to have evolved from symbiosis (known as the serial endosymbiotic theory, or SET) between early eukaryotic cells and aerobic bacteria in an event that occurred over a billion years ago [33, 34]. Often referred to as bacteria-size organelles (2–4 *μ*m), mitochondria vary not only in their number per cell but in their localization, size, shape, and features, adapting their function to the needs of the cell at hand. For example, while it has been estimated that hepatocytes in mammals contain roughly 800 mitochondria per cell, mammalian oocytes are estimated to contain over 100,000 [35]. Nearly 200,000 copies of the mitochondrial genome can be found per oocyte on average, with content affecting fertilization capacity [36].

Mitochondria possess two membranes: the inner mitochondrial membrane (IMM) that forms the cristae and the outer mitochondrial membrane (OMM). Central to the organelle are also the aqueous compartments, the mitochondrial matrix, and the intermembrane space. Tight regulation of ion flow between these distinct pockets is essential for the ETC's core functions. In animals, the mitochondrial genome is comparatively small, averaging about 16,500 base pairs and equaling 16,569 base pairs in humans [37]. It is devoid of introns, with the exception in select lower animals such as sea anemones [38]. In comparison, the mitochondrial genome of plants has evolved in a remarkably opposite direction, amassing much larger sizes in the range of 15 kbp to 2.4 Mbp and containing numerous processing elements, including introns [39]. It is apparent that despite divergent evolutionary tracks, mitochondria are essential to support increased energy demand under certain conditions such as exercise, and controlled regulation is critically needed for multicellular life to exist.

The ETC utilizes electrons derived from food molecules that enter the chain at complexes I and II. Both complexes transfer the electrons to ubiquinone from their substrates NADH and FADH_2_, respectively. These electrons are subsequently transferred to complex III, where they are used to reduce two molecules of cytochrome *c*. Cytochrome *c* will then shuttle these electrons to COX, which terminates the chain by transferring the electrons to dioxygen, generating water. COX is similar to complexes I and III in that electron transport is coupled to the pumping of protons from the mitochondrial matrix to the intermembrane space, contributing to the formation of the electrochemical gradient, of which the mitochondrial membrane potential (Δ*Ψ*_m_) constitutes the major part in animals. This force drives ATP synthase in its synthesis of ATP from ADP and inorganic phosphate [40]. Acting as a rotary motor, ATP synthase uses the combined proton motive force generated from the other complexes to generate rotational and eventually chemical energy by changing conformation to combine a phosphate molecule with ADP to form ATP [41].

Until recently, characterization of the ETC has largely been based around a random-collision model, where individual components and substrates interact as a function of concentration and chance [42, 43]. There has been a growing trend towards studying the ETC as a solid-state system, a phenomenon known as supercomplexes. With the exception of succinate dehydrogenase (complex II) and ATP synthase, the remaining components of the ETC have been shown to associate with one another with varying stoichiometries of complexes I, III, and IV [44–47]. Evidence for the formation and stabilization of supercomplexes has largely been based around the isolation of complexes using two-dimensional blue native gel electrophoresis (2D-BN-PAGE) [48, 49]. Recently, new factors have been identified to be important for the formation and modulation of supercomplexes, including isozymes and assembly factors of COX [50–52].

## 2. Composition of Cytochrome *c* Oxidase

COX is the terminal enzyme of the mitochondrial respiratory chain. Mammalian COX from bovine heart was crystalized as a 13-subunit, homodimeric enzyme [53]. However, it contains at least one more less tightly bound subunit in stoichiometric amounts, NDUFA4 [54], which was initially thought to be a subunit of complex I. COX is one of only four mitochondrial complexes that are encoded by both the nuclear and mitochondrial genomes and that are all components of the OxPhos process (i.e., complexes I, III, IV, and V but not complex II, which is encoded entirely by nuclear DNA). Bigenomic enzymes are unique in that their regulation requires tight coordination between the nuclear and mitochondrial genomes. For the sake of clarity and consistency, in this review, the nomenclature assigned to each of the subunits by Kadenbach et al. will be used [55]. Of these subunits, the three largest subunits (COX I, II, and III) are encoded by the mitochondrial DNA; the remaining 11 subunits (COX IV, Va, Vb, VIa, VIb, VIc, VIIa, VIIb, VIIc, VIII, and NDUFA4) are encoded by the nuclear genome and play critical roles in energy metabolism and regulation. With the exception of subunits Va and Vb, which are bound to the matrix side, and subunit VIb, which faces solely the intermembrane space, each of the remaining subunits contains a hydrophobic transmembrane region. Through the contribution of four electrons transferred via cytochrome *c* and four protons channeled from the mitochondrial matrix, it is capable of reducing dioxygen to water.

Of the mitochondrial-encoded subunits, subunits I and II carry out the catalytic reaction. They are the largest and third largest subunits of the holoenzyme, respectively. Subunits I and III are highly hydrophobic in nature and contain multiple transmembrane domains, which suggests a rationale for being encoded in the mitochondria, thereof avoiding complicated protein import from the cytosol and possible aggregation. In contrast, the relatively smaller and more hydrophilic nature of the nuclear-encoded subunits allows for posttranslational localization to the mitochondria. Catalytic subunits I and II contain prosthetic metal groups. Subunit I contains both a low-spin heme *a* redox center and a high-spin Cu_B_-heme *a_3_* binuclear center, while subunit II contains a Cu_A_ redox center formed by two copper ions. Of these redox sites, the Cu_A_ site is responsible for the initial step of the catalytic cycle by accepting the electrons transferred from cytochrome *c*, which then reduces the heme *a* site in COX I. These electrons are then subsequently transferred to the Cu_B_-heme *a_3_* site, where molecular oxygen binds and is reduced to water [56]. Molecular inhibitors such as CO, NO, cyanide, or azide bind to the Cu_B_-heme *a*_3_ center, preventing the binding of oxygen and stopping the enzyme's catalytic action.

The movement of protons is accomplished through two proton uptake pathways, known as the D and K channels [57–59], named after the conserved residues located at the matrix side and the opening of the proton channels. These two channels deliver protons required for the water formation reaction as well as the pumping of protons. The D and K channels are well understood and demarcate the lower half of the proton network from the matrix up to the heme groups located near the middle of the membrane including the oxygen binding site. However, the proton exit pathways and the precise proton pumping mechanism remain unknown despite a wealth of proposed models [58, 60–62]. A third channel, referred to as the H channel, was proposed based on the bovine COX structure and mutational analyses [63–65]. However, mutational studies with the corresponding amino acids in bacterial COX from *Paracoccus denitrificans* questioned the presence of this pathway at least in the bacterial enzyme [66].

## 3. Allosteric and Posttranslational Regulation of Cytochrome *c* Oxidase

As expected of an enzyme with critical functions in membrane potential homeostasis and control of electron flux, COX is tightly controlled through multiple regulatory processes including allosteric regulation and posttranslational modifications. Although it is not a focus of the current article, a few select examples will be briefly discussed.

In the presence of ADP, the binding affinity for cytochrome *c* to COX is increased by fivefold as compared to that of ATP, indicating that enzyme activity is modulated allosterically by the ATP/ADP ratio [67–69]. Unsurprisingly, COX is also regulated through phosphorylation of serine/threonine and tyrosine residues [70]. To date, detection of in vivo phosphorylation sites through mass spectrometry has yielded 18 different targets [71], though the specific functions of most remain unknown. One such modification that has been characterized is the inhibitory phosphorylation of tyrosine 304 of COX I, in a cAMP-dependent manner [72]. This modification was later shown to be also stimulated by TNF*α* in the liver through an inflammatory cascade, resulting in diminished COX function and ATP levels [14]. This phosphorylation was then proposed to be an underlying mechanism of disease conditions as seen in acute inflammation or sepsis, in which oxygen utilization is impaired despite oxygen availability, a phenomenon called cytopathic hypoxia. Serine 441 on the same subunit was suggested to act as a functional toggle for the allosteric inhibition of COX by ATP through phosphorylation, but subsequent mass spectrometry analysis was unable to detect this modification [73]. Furthermore, on subunit IV-1, serine 58 was suggested through targeted mutational analysis as capable of performing this function, and protein kinase A (PKA) was proposed to phosphorylate this site and enable ATP to inhibit the enzyme allosterically [74]. However, experimental evidence that this site can be phosphorylated, for example, through mass spectrometry, still has to be provided. It should also be noted that this cAMP-dependent phosphorylation takes place on the matrix side of COX and is distinct from the indirect cAMP-dependent phosphorylation on tyrosine 304 [72], which occurs in the mitochondrial intermembrane space and cannot be mediated by PKA since it does not target tyrosine residues. See [4, 71] for comprehensive reviews of this literature.

As a side glance, COX can also be externally regulated through application of near-infrared light (IRL). COX contains two copper centers that are involved in enzyme catalysis and have been shown to function as the photoacceptors for IRL [75, 76] because Cu^2+^ broadly absorbs IRL in the range of 700–1000 nm as can be seen in the COX spectrum [77]. IRL was proposed to activate COX leading to health benefits in several studies including improving cognitive function in humans, increasing cell survival in cultured neurons in vitro after poisoning of COX with inhibitor potassium cyanide, and improving wound healing, just to name a few [78–82]. Modulation of COX activity in conditions of mitochondrial dysfunction seems to be an interesting area worth exploring for clinical applications, in particular because of the noninvasive nature of the treatment.

## 4. Synthesis and Assembly of Cytochrome *c* Oxidase

The assembly of COX is a complex, tightly-regulated process with a large number of auxiliary components. To date, over 30 gene products have been identified that are solely involved in the biogenesis of the holoenzyme [83]. These products include a variety of participants, ranging from translocases, translational activators, and molecular chaperones to metallochaperones and enzymes involved in the biosynthesis of heme A [84]. The earliest points of translation and assembly have best been studied in the yeast *Saccharomyces cerevisiae*, where the translational activators Mss51 and Pet309 are responsible for the early regulation of COX I transcription and translation [85–90]. In mammals, however, mitochondrial mRNAs contain minimal 5′-UTR regions for translational activators to bind to, indicating that regulation of COX I translation may be controlled through an alternative pathway [91]. The gene TACO1 has been hypothesized to fulfill this role, as patients with mutations in the gene suffer from a progressive form of Leigh syndrome alongside reduced translation of COX I [92]. In mice, a TACO1 missense mutation was linked with reduced COX I translation, deficit in total COX levels, and late-onset mitochondrial dysfunction contributing to visual deficit and motor impairment [93]. In general, much of what is known of mammalian COX assembly is gleaned from investigation of mitochondrial diseases and the enzymatic deficiencies presented [94–96]. The majority of reported COX-associated disease has been attributed to mutations in assembly factors and early chaperones [97]. However, the nuclear-encoded subunits, particularly those with tissue- or condition-specific isoform expression, have also emerged as disease-causing or likely disease-causing candidate genes in COX deficiencies (see Table 1). The fact that mutations in the nuclear-encoded subunits of COX are very rare highlights the subunits' importance for COX function, regulation, and stability.

**Figure 1:**
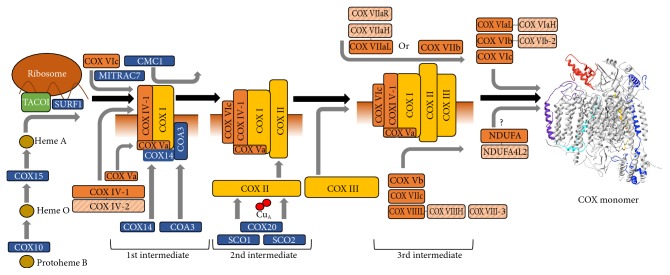
The early assembly of cytochrome *c* oxidase. Assembly of COX begins with translation of mitochondrial RNA with mitochondrial ribosomes and proceeds in a series of intermediates. The inner mitochondrial membrane is shown in conjunction with the partially assembled enzyme intermediates, with the bottom region denoting the matrix side. TACO1, which is involved in RNA binding, is shown in green, assembly factors are in blue, and mitochondrial-encoded subunits (COX I, COX II, and COX III) are in yellow. Nuclear subunits are indicated in orange, with isoforms grouped and denoted with a striped pattern in light orange.

Assembly of COX is a highly regulated process which integrates cytosolic and matrix protein synthesis of nuclear- and mitochondrial-encoded subunits (Figure 1). Even the first step, synthesis of COX subunit I, is controlled, via interaction of COX I mRNA containing ribosomes with COX assembly factors to synchronize with the influx of nuclear-encoded subunits [98]. COX assembly begins with the translocation of COX I to the membrane, followed closely by the association of subunits IV and Va [99]. The twin-CX_9_C intermembrane protein CMC1 stabilizes COX I, in tandem with the COA3-COX14 early intermediate, prior to the incorporation of any other COX subunits. *CMC1*-knockout cells showed a 30% reduced basal respiratory rate, accumulation of COX assembly intermediates, and very low to undetectable levels of COX in I + III_2_ + IV_n_ supercomplexes [100]. Translation of COX I mRNA is completed through integration of the critical heme A moiety. Biosynthesis and insertion of heme A into COX I require the assembly factors COX10 and COX15, which are involved in maturation of the protoheme through several stages, as shown from multiple studies of yeast mitochondria [101–103]. These assembly factors have been implicated in COX assembly in mammals primarily through studies linking their defect to mitochondrial disease, specifically Leigh syndrome and cardiomyopathies [104–108]. The assembly protein SURF1 has been a subject of investigation for its known role in early COX biogenesis, as complexes lacking the protein stall in assembly with partial holoenzyme products containing only subunits I, IV, and Va in humans [109]. Mutations in the *SURF1* gene in humans cause Leigh Syndrome [110], a severe neurodegenerative condition with early lethality due to COX deficiency. In stark contrast, mice that are null for *Surf1* live longer than control mice despite lower COX activity [111]. The knockout of SURF1 has also been linked to increased oxidative stress and induction of the mitochondrial unfolded protein response [112, 113]. Furthermore, despite increased reactive oxygen species (ROS) production, *Surf1*-knockout mice have shown increases in glucose metabolism, memory, and blood flow in the brain [114]. Analysis of mouse fibroblasts with a homozygous *Surf1* knockout showed only marginal differences in assembly products compared to that of wild-type mouse fibroblasts, indicating that SURF1 may vary in its importance in assembly in a species-specific manner [115, 116]. Since *Surf1* knockout in mice results in a much milder phenotype to that seen in patients with point mutations, an alternative interpretation would be that the mutant protein products may still interact and bind to COX assembly intermediates. This could result in assembly pausing and accumulation of dysfunctional COX intermediates, further enhancing mitochondrial dysfunction. Indirect evidence of such a scenario was seen in three cell lines from Leigh syndrome patients, which showed a higher running band in a Western blot with a COX subunit IV antibody [117]. Since denaturing conditions were used in this experiment, it is conceivable that COX subunit IV forms a covalent intermediate with SURF1 (or another protein acting in concert with SURF1), but mutation or truncation of SURF1 prevents the release of COX subunit IV. Such COX subunit IV-SURF1 intermediates would not be possible in the knockout, possibly explaining the mild phenotype. Future work should further characterize the COX subunit IV containing covalent intermediates with the potential of revealing the molecular mechanism of SURF1's chaperone function.

The human analog of the yeast protein COA3, known as CCDC56 or hCOA3, has been shown to stabilize subunit I in the process of assembly and is critical for proper COX function [118]. Another study identified the mitochondrial chaperone MITRAC7 in the early assembly of COX [119]. The authors concluded that it associates with a COX I/COX IV/COX VIc intermediate to stabilize it before progressing to the next stage of assembly (Figure 1). Knockouts of MITRAC7 show increased COX I turnover and reduced biogenesis, whereas overexpression leads to accumulation of the early intermediate and concurrent reduction in complete COX assembly.

Assembly continues with association of the mitochondrial-encoded COX subunit II into a transient intermediate [120]. Continued assembly requires incorporation of copper into the catalytic core before the mature holoenzyme can be established. This is accomplished through action of the metallochaperones SCO1 and SCO2; both have independent functions in incorporating copper into the Cu_A_ site of COX II [121–123]. The COX assembly factor COX20, also known as FAM36A, is an integral part of this process—COX20 stabilizes COX II in the process of copper insertion, and its absence results in inefficient incorporation into assembly intermediates [124]. Mutation of this gene results in ataxia and muscle hypotonia as a consequence of COX deficiency [125]. After insertion of heme *a* and copper, the COX I/COX II/COX IV/COX Va intermediate associates with COX3 and subsequently incorporates the remaining nuclear subunits in a relatively swift manner. Very little is known about the exact order of incorporation, though hypotheses may be drawn from the physical relationship of the individual subunits. It has been shown that the immature enzyme incorporates subunits Vb, VIc, and VIIa or VIIb, VIIc, and VIII, before subsequent incorporation of VIa, VIb, and whichever of VIIa or VIIb that remains [94, 126]. In addition, the time point of the incorporation of NDUFA4 remains unknown. After full assembly of the 14 subunits into a monomer, the holoenzyme stabilizes as a functional dimer [53, 127].

Although many of the unique interactions surrounding COX assembly are still unclear, the molecular mechanisms responsible for degradation or replacement of the individual subunits remain even more obscure. Regulation of COX subunit transcription at the mRNA level has been explored in the context of temperature fluctuations in goldfish. It was concluded that individual subunits are universally controlled at the transcription level, but degradation rates may differ and be responsible for differential transcript levels in cold acclimation [128]. Recently, the mitochondrial ATPase lactation elevated 1 (LACE1) was investigated for its role in degradation of COX, based on sequence homology with the yeast ATPase Afg1, which serves a similar role [129, 130]. It was found that LACE1 directly interacts with subunits IV and Va and is responsible for proteolysis of excess subunits IV, Va, and VIa [131].

The hypothesis that the function of COX may be controlled by tissue-specific isoform expression was first proposed by the Kadenbach group [132] and later confirmed by many others. Small differences in molecular weights of subunits harvested from multiple mammalian species and tissues led to the suggestion that there may be different isoforms present. Given the distinct energy demand and response to external regulators such as hormones and second messengers in highly specialized organs such as the heart, kidney, liver, skeletal muscle, lung, testes, and brain, it is not surprising that divergent isoforms have evolved to accommodate these conditions. It is surprising, however, that in mammals, only COX and its partner cytochrome *c* have tissue-specific and developmentally regulated isoforms, whereas none have been reported for the other OxPhos complexes. An explanation for the requirement of a fine-tuned regulation in COX may be as follows: it was suggested that the reaction catalyzed COX and cytochrome *c* is the rate-limiting step of the ETC in intact cells and tissues under physiological conditions [133–137]. The free energy released in this reaction (ΔG^o^' = 100 kJ/mol) is about twice as high compared to that in complexes I and III [138]. This makes it an essentially irreversible reaction, which may explain why the terminal step of the ETC is a particularly important target for regulation. Thus, one central purpose of this article is to highlight the regulatory features of tissue-specific isoform expression of COX subunits in mammalian systems. The intent is to present this topic from two perspectives: the regulatory elements that control expression on a genetic level for the induction of isoforms triggered by certain conditions such as hypoxia and the effect of isoform expression and regulatory function within the context of the COX holoenzyme itself. Investigating the nature of these features within their given tissue context including tissue-specific energy requirements will allow elucidation of potential hypotheses for their existence, summarized in Figure 3, and connect tissue-specific isoform expression with varying properties of COX.

## 5. Isoforms of Cytochrome *c* Oxidase Subunits

Given the critical role of COX in regulating oxygen consumption and ATP production, it is of no surprise that isoform expression is regulated through multiple mechanisms. It can be broadly sorted into two overlapping categories: hypoxia-induced and development-induced. The first category comprises isoforms that are differentially expressed through oxygen tension, including subunit IV-2 and the newly identified subunit NDUFA4. These subunits carry a ubiquitously expressed isoform alongside an isoform preferentially induced under hypoxic conditions and expressed only in certain tissues. It is important to note that in addition to regulation via oxygen, COX subunit IV-2 is also developmentally induced as discussed below. The second class of COX isoform pairs can be described as development-specific isoforms, including isoforms of subunits VIa, VIb, VIIa, and VIII. A subset of these, subunits VIa, VIIa, and VIII, contain a “liver-type” (L) and “heart-type” (H) isoform. During maturation, in particular after birth, the liver-type isoforms are switched to heart-type in the heart and skeletal muscle. Finally, subunit VIb has a somatic- and testes-specific isoform. Note that roman numbers are used to refer to the protein whereas standard numerals and italics are used to refer to the gene with all capital letters referring to the human gene.

### 5.1. Oxygen-Regulated Isoforms

#### 5.1.1. Subunit IV

**Figure 2:**
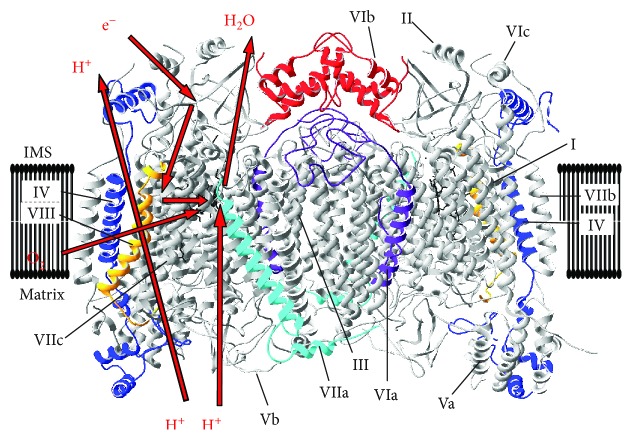
Structure of cytochrome *c* oxidase. Crystallographic data of cow heart COX [53] were processed with the program Swiss-PDBViewer 4.1. COX subunits that have isoforms are highlighted in color: IV, blue; VIa, purple; VIb, red; VIIa, cyan; and VIII, yellow. Left monomer: electron flow from cytochrome *c* to Cu_A_, heme *a*, heme *a_3_*/Cu_B_ and molecular oxygen and concomitant proton pumping are schematically shown. Note that COX subunit NDUFA4, which is less tightly bound to COX and lost during purification, is not shown.

The largest of the nuclear-encoded subunits, COX subunit IV, is located adjacent to the catalytic subunits, containing numerous contact sites with subunits I and II [53] (Figure 2). This pivotal location allows the subunit to play a major role in regulation of overall COX activity. As discussed below, COX IV has been shown to contain a conserved ATP binding pocket on the matrix side, allowing for allosteric inhibition of COX activity at high ATP/ADP ratios [74, 139, 140]. In *S. cerevisiae*, the corresponding COX subunit (subunit V in yeast nomenclature) is expressed as two isoforms, COX Va and COX Vb, which are expressed in varying amounts dependent on the oxygen concentration. COX Va is preferentially expressed in normoxic conditions, while COX Vb is induced under hypoxia, allowing for control of enzyme function dependent on oxygen concentration [141–143]. The hypoxic isoform Vb has a higher turnover rate and intramolecular electron transfer rate than isoform Va contained in yeast COX [144]. It was proposed that mammalian COX IV serves a similar purpose to that of yeast COX V, with differential expression of two isoforms in response to local oxygen conditions [140, 145].

The principal isoform, mammalian COX IV-1, is ubiquitously expressed in all tissues in vertebrates. It has been shown to be a required component for COX biogenesis, coordinating the assembly of the holoenzyme alongside the mitochondrial-encoded subunit I [126]. This subunit has been shown to be responsible for modulating COX activity through allosteric regulation—ATP and ADP are capable of binding to COX through subunit IV, resulting in fine-tuned control of respiration [67, 69, 139]. This ATP-mediated inhibitory effect was proposed to require phosphorylation of the subunit by PKA [146, 147].

Analogous to yeast COX, there is a second isoform of COX subunit IV in animals, which is expressed differentially in response to changes in oxygen concentration. Interestingly, the COX IV-1/IV-2 isoform pair found in mammals today arose by a gene duplication event about 320 million years ago [140], earlier in evolution compared to that of the origin of the other isoform pairs and at a time when atmospheric oxygen concentrations fluctuated dramatically [148], suggesting a possible adaptation to varying oxygen levels. The second isoform, named COX isoform IV-2 (*COX4-2* or *COX4I2* for the gene), was first discovered in tuna fish and found to share 56% sequence homology with COX4-1 at the protein level [149]. Following these studies, *COX4-2* was identified and characterized as a component of COX expressed in mammals including humans [140]. The precursor peptides of *COX4-1* and *COX4-2* are similar in length with 169 and 171 amino acids, respectively. The two isoforms share only 44% nucleotide homology averaged across mammalian species, while *COX4-2* itself shows high sequence homology of 78% between the species analyzed [140]. Quantitative PCR performed on rat tissues revealed that *COX4-2* is primarily lung-specific, showing similar expression levels compared to the ubiquitous *COX4-1* isoform, followed by expression in the placenta [150] and minor expression in the heart (~8%) and brain (~4%) [140]. Similar results have been found in mice, showing virtually no expression in liver or pancreatic tissue [151]. *COX4-2* is also developmentally regulated and strongly induced after birth in human lung [140], as discussed for other COX isoforms in the next section.

One interesting feature of COX IV-2 is that it contains three unique cysteine residues, one within the transmembrane region and two on the matrix side, near the proposed ATP binding site for allosteric regulation [140]. The ubiquitous COX IV-1 contains no cysteine residues. This suggests that *COX4-2* may incorporate redox signaling as part of its function, given that the twin cysteines on the matrix side are close enough to potentially form a disulfide bond. In addition, the internal cysteine residue may interact with other proteins or be modified posttranslationally in response to redox changes within the membrane.

The biochemical and physiological effects of incorporating COX IV-2 in the COX holoenzyme have begun to be characterized, providing necessary insight into the functional features of this isoform. Isolated COX from cow lung, containing COX IV-2, was shown to have about twofold increased activity compared to liver COX, which does not contain COX IV-2 [151]. In order to study the effects of COX IV-2 in vivo, a mouse model containing a knockout of *Cox4-2*, created by deletion of exons 2 and 3, was established [151]. It was demonstrated that COX activity was similarly modulated by knocking out *Cox4-2*, as lung COX from the wild-type mice showed twofold increased activity compared to that from the knockouts. In addition, ATP levels were reduced by 29% in the knockout mice versus the controls, suggesting that COX activity modulates cellular energy levels by acting as a bottleneck for ETC flux. The physiological consequences of *Cox4-2* expression, or lack thereof, have been demonstrated with varying levels of severity. The knockout mouse model was studied through a detailed functional screen with a focus on lung function, and it was discovered that lack of *Cox4-2* has significant ramifications. *Cox4-2-*knockout mice showed reduced airway responsiveness, with 60% reduced *P*_enh_ and 58% reduced airway resistance when challenged with methacholine [151]. This finding in the knockouts of decreased ability for the airways to constrict—a process that requires energy—may be explained by decreased energy levels found in the lungs of the knockout mice. Furthermore, the mice showed a consistent, chronically deteriorating lung pathology and presented with lung inflammation, fibrosis, the recruitment of macrophages, and the development of Charcot-Leyden crystals, which are hypothesized to be formed from the products of eosinophil breakdown [152, 153].

Given the profound effect of isoform expression on the activity of COX, it is likely that expression of *COX4-2* presents an intricate and complex story of transcriptional regulation. We showed previously that *COX4-2* is regulated by a novel oxygen responsive element (ORE) located in the proximal promoter of the gene. Using reporter gene analyses, expression of *COX4-2* was shown to be maximal at 4% O_2_ with an about threefold induction compared to normoxia [154]. The highly conserved 13 bp ORE was later shown via a yeast one-hybrid screen to interact with transcription factors MNRR1, RBPJ, and CXXC5 in a complex manner to regulate the expression of *COX4-2* [155]. Of the three factors, the protein MNRR1 (mitochondria nuclear retrograde regulator 1), also known as CHCHD2, has some novel features. It has been found to function as a biorganellar signaling molecule to communicate between the mitochondria and the nucleus. It directly binds to and modulates COX activity when localized to the mitochondria, and it has been shown to be present in the nucleus, where it functions as a transcriptional regulator of *COX4-2* [156]. In this model, RBPJ and MNNR1 work in tandem to activate transcription of *COX4-2*, while CXXC5 functions as a repressor, allowing up- or downregulation of gene expression depending on signals or stresses, such as a change in the oxygen concentration. Interestingly, analysis of HEK293 cells exposed to 4% oxygen showed a significant increase in MMNR1 protein levels, supporting the idea that *MMNR1* and *COX4-2* share regulatory features under hypoxic stress [155]. In fact, the *MNRR1* promoter contains its own ORE and its expression is thus under autoregulatory transcriptional control. Oxygen regulation of *COX4-2* is a unique phenomenon for mammals, as nonmammalian species including several fish and reptiles do not show any changes in transcription levels in response to oxygen concentration [157].

*COX4-2* was also proposed to be regulated through the HIF-1 at very low oxygen concentrations [158]. Here, 1% oxygen was sufficient to induce and stabilize HIF-1 and in turn upregulate expression of a *COX4-2* reporter gene. In addition, the mitochondrial protease LON was induced, which is proposed to be required for the degradation of COX IV-1 and insertion of COX IV-2. It is not fully clear whether or not 1% oxygen is physiologically relevant in regard to the lung, given the much higher exposure to oxygen taking place in this organ, but it may well be encountered during pathological conditions such as chronic obstructive pulmonary disease. However, the role of HIF-1 in regulating *COX4-2* remains controversial, since a recent study, using HIF-1 wild-type and knockout mouse embryonic fibroblasts, concluded that the oxygen-dependent regulation of *COX4-2* is not mediated by HIF-1 [159].

In addition to expression in the lung, *COX4-2* has also been detected at lower levels and studied in other tissues and tissue models. Under toxic conditions applying complex II inhibitor 3-nitropropionic acid [160] or anoxic conditions [161], *COX4-2* was shown to be upregulated in cortical astrocytes about threefold, confirming that the gene responds to stress and oxygen concentration. However, it remains to be shown what the basal ratio of the two isoforms is in COX of astrocytes and, consequently, if an induction of *COX4-2* can result in a significant change in the composition of the COX protein pool towards an enzyme pool containing more COX IV-2. In addition, *COX4-2* expression was found to be negatively correlated with cancer aggressiveness in gliomas, while those expressing only *COX4-1* were found to be more aggressive and capable of cell growth [162]. Finally, mutation of *COX4-2* in humans has also been linked to pancreatic pathology, as an E138K mutation was identified as the driver of exocrine pancreatic insufficiency, dyserythropoietic anemia, and calvarial hyperostosis in a clinical investigation of four patients [163]. As *COX4-2* is predominantly expressed in the lung and has not otherwise been reported as a pancreatic gene, this data may suggest that deficiency of certain organs or cell types where *COX4-2* is expressed may result in diseases of other organs, potentially during development. Alternatively, *COX4-2* may be expressed in a minor pancreatic cell type. Another heterozygous missense mutation was reported in a patient with COX deficiency but not functionally confirmed [164].

#### 5.1.2. NDUFA4

The latest subunit to become a recognized stoichiometric component of COX is NADH dehydrogenase (ubiquinone) 1 alpha subcomplex 4, also known as NDUFA4. This nuclear-encoded transmembrane protein was originally described as one of the 45 subunits of complex I [165]. However, recent advances in gene expression analysis have shown that expression patterns of *NDUFA4* diverge from those of other nuclear-encoded complex I subunits, potentially highlighting its role in other complexes [166]. In support of this concept, Balsa and colleagues recently presented data showing that the subunit is instead a functional and stoichiometric component of COX [54]. This was done by using the mild detergent digitonin to solubilize the mitochondrial membrane, allowing isolation of intact COX while preserving protein-protein interactions with high integrity. As a component of complex IV, NDUFA4 could prove to be a useful target in discerning the genetic nature underlying diseases caused by mitochondrial energy deficit, such as Leigh syndrome and similar COX deficits. Pitceathly and colleagues recently provided this link by examining a consanguineous family afflicted with isolated COX deficiency [167]. It was found that rather than mutations in traditionally associated COX subunits, the family was affected by homozygous donor splice site mutations in *NDUFA4*, resulting in protein loss-of-function, with the further suggestion that families suffering from unexplained COX deficiency should be screened for NDUFA4 mutations.

There is an isoform of NDUFA4 known as NDUFA4L2, whose functions have only been studied in the context of NADH dehydrogenase up to this point. Under hypoxic conditions, *NDUFA4L2* transcription has been found to be upregulated through HIF-1*α* stabilization, where it was the only gene categorized as a component of complex I to be responsive [168]. Expression of *NDUFA4L2* was found to reduce oxygen consumption by 42% under hypoxic conditions in HeLa cells, compared to 27% reduction when *NDUFA4L2* expression was silenced by 80%. Transient overexpression under normoxic conditions was found to have a similar effect, reducing oxygen consumption by 20%. Silencing of *NDUFA4L2* was also found to increase ROS production as measured by H_2_-DCFDA and MitoSOX, as well as to increase the mitochondrial membrane potential. Interestingly, under hypoxic conditions, complex I activity was reduced by 20% in *NDUFA4L2* knockdowns while complex IV activity was not affected. Given that NDUFA4 was only recently established as a component of complex IV, this may indicate that NDUFA4 and its alternative isoform instead play a role in supercomplex formation or association and may attenuate complex I activity through some unknown interaction in hypoxic conditions. A further study on both the ubiquitous and the hypoxia-induced isoforms is necessary to establish the specifics of this potentially critical regulatory relationship.

Although the specific functions of NDUFA4L2 remain elusive, its clinical significance has recently been underscored in a number of studies relating to several types of cancer. *NDUFA4L2* expression has been shown to be highly induced in clear-cell renal cell carcinoma, whereas normal kidney shows no significant expression [169]. In addition, expression levels were positively correlated with stage, with increasing expression in later-stage renal cancer. Cell culture models knocking down *NDUFA4L2* showed impaired proliferation and colony-forming capacity. Additionally, metabolic pathways were shifted away from the pentose phosphate pathway, with downregulation of key enzymes involved and upregulation of TCA cycle pathway members, indicating a shift towards glycolytic growth. A separate study associated an increase in *NDUFA4L2* expression with poor prognosis in patients with colorectal cancer [170]. Overexpression of *NDUFA4L2* was found in 84% of colorectal cancer tissue samples, compared to about 25% of adjacent normal tissue. Kaplan-Meier statistical analyses for overall survival and tumor-free survival both showed reduced survival rates in patients with *NDUFA4L2* overexpression versus those with low or undetectable expression. Similarly, using human hepatocellular carcinoma cell lines, *NDUFA4L2* was found to be dramatically overexpressed when exposed to hypoxia, as a result of HIF-1*α* induction [171]. A comparison of 100 cases of human hepatocellular carcinoma revealed that 71% showed overexpression of *NDUFA4L2* and had a lower 5-year overall survival rate as compared to controls. Knockdown of *NDUFA4L2* suppressed proliferation of tumors in a mouse model and increased ROS production as measured by H_2_-DCFDA fluorescence. Notably, suppression of HIF-1*α* through the pharmacological inhibitor digoxin resulted in suppressed tumor proliferation without affecting mouse bodyweight, indicating that targeting HIF-1*α* may be a valuable therapeutic tool in cells overexpressing *NDUFA4L2*.

### 5.2. Developmentally Switched Tissue-Specific Isoforms

COX subunits VIa, VIIa, and VIII have multiple tissue-specific isoforms expressed in mammals. During heart and skeletal muscle development, there is an isoform class switch from the liver (nonmuscle form) to the muscle isoform. The liver-type isoforms are thought to be ubiquitously expressed, while the heart-type subunits (VIaH, VIIaH, and VIIIH) are expressed in the heart and skeletal muscle [172]. In rats, an increase in COX VIaH and VIIIH and a concurrent decrease in liver-type isoforms were observed shortly after birth [173, 174]. All these isoforms are products of separate genes located on different chromosomes rather than products of alternative splicing of the same gene.

#### 5.2.1. Subunit VIa

The gene duplication event that gave rise to nowadays mammalian subunit VIa isoform pair occurred about 240 million years ago [175]. The protein looks somewhat like an S-shaped hook, connects the COX monomers in the membrane region of the enzyme [53], and therefore stabilizes the COX dimer (Figure 2).

The liver isoform of subunit VIa was indirectly concluded to modulate proton pumping efficiency of COX (i.e., the proton to electron stoichiometry). COX purified from cow kidney tissue, a tissue that similarly to that of the liver expresses the liver-type isoforms and reconstituted into vesicles, showed a 50% reduction in the proton to electron stoichiometry in the presence of the fatty acid palmitate whereas other fatty acids showed no effect [176]. The authors proposed that such an uncoupling mechanism could contribute to thermogenesis in warm-blooded animals.

The heart enzyme, containing COX VIaH, did not show a change of the proton to electron stoichiometry in the presence of palmitate. However, this isoform binds to allosteric regulator ADP on the matrix side of the enzyme, increasing enzyme activity, an effect that could be prevented in the presence of a COX VIaH-specific antibody [177]. In the presence of very high ATP/ADP ratios, COX VIaH mediates a 50% decrease in the proton to electron stoichiometry [178]. Similar to the effect of palmitate on COX VIaL, the authors proposed that ATP-mediated uncoupling contributes to thermogenesis during periods of physical inactivity with high ATP/ADP ratios in muscle, such as during sleep.

In 2002, the first COX subunit isoform-knockout model was introduced, in which the gene encoding isoform VIaH was deleted in mice [179]. A surprising finding was that despite reduced COX activity, myocardial ATP levels were similar to those of controls under basal conditions. However, since the mice developed cardiomyopathy over time, it is clear that COX VIaH is required for proper COX function and likely more so under increased performance conditions, such as strenuous exercise, which have not been studied yet.

#### 5.2.2. Subunit VIb

COX subunit VIb occurs as a somatic- (COX VIb1) and testes-specific isoform (COX VIb2). This subunit is unique among the nuclear-encoded subunits of COX in that it is solely located on the mitochondrial intermembrane space side of the holoenzyme, connecting the COX monomers [53] (Figure 2). This subunit can be separated from the core enzyme through treatment with the detergent dodecylmaltoside, resulting in twofold increased enzyme activity [180]. These kinetic alterations suggest that subunit VIb downregulates COX activity and that removal of this subunit may monomerize the holoenzyme. Accordingly, it has been proposed that COX VIb may be responsible for the cooperative activity of the two COX monomers once assembled into the dimer. Mutations in COX VIb1 have been implicated in disease phenotypes associated with COX deficiency. A missense mutation in a conserved arginine residue, R19H, resulted in severe infantile encephalomyopathy [181]. A second study found that alteration of the same residue to a cysteine, R19C, resulted in encephalomyopathy, hydrocephalus, and hypertrophic cardiomyopathy [182].

More recently, a second isoform of the subunit, COX VIb2, was discovered in human, mouse, rat, and bull [183]. Interestingly, the *COX6b2* gene was found to be exclusively expressed in the testis; in mouse and rat, it is the only transcript present, while in humans and bulls, both isoforms are present. In rodents, a testis-specific isoform of cytochrome *c* is also present [184]. This suggests that there may be unique energy demands of spermatozoa that are addressed through isoform expression of ETC components. Given the function of COX VIb1 to downregulate COX activity, equipping the enzyme with a subunit isoform in sperm may provide a unique target for cell signaling during sperm activation, to activate OxPhos when energy is needed for movement.

#### 5.2.3. Subunit VIIa

Subunit VIIa (Figure 2) has three isoforms. Similar to subunit VIa, there is a liver-type and heart-/skeletal muscle-type isoform (note that nomenclature of the heart-/muscle-type isoform genes for subunits 6a and 7a is reversed, i.e., *COX6a1* and *COX7a2* are the liver-type genes and *COX6a2* and *COX7a1* are the heart-type genes).

The expression of *COX7a1* was determined by Northern blot to be present strongest in adult mouse heart and skeletal muscle, with minor hybridization present in adult kidney and lung tissue [173]. For *COX7a2*, expression was detected in all adult and fetal tissues, which included those of the heart and skeletal muscle. The fact that the liver-type mRNAs are present but not (humans) or not highly (rodents) translated in the heart and skeletal muscle can be explained by posttranscriptional regulation. In tissues that express the liver-type isoform proteins, translation is assisted by the presence of auxiliary proteins, which bind to the 3′-untranslated regions of the mRNAs [185, 186].

Similar to the knockout approach of the heart-type isoform of subunit VIa, we later generated a whole-body mouse knockout of *Cox7a1* [187]. The knockouts were normal in appearance with morphologically normal mitochondria. However, their heart mitochondria showed a 15% reduction in COX levels, a 32% reduction in COX activity, and a 29% reduced respiratory control ratio, which is a measure of mitochondrial coupling. The heart size was increased significantly and the heart weight was 15–20% higher compared to that of controls. In addition, as demonstrated by echocardiography, the hearts of the knockout mice showed reduced systolic and diastolic function.

Analysis of the skeletal muscle in the Cox7aH knockouts also revealed dysfunction with over 60% reduced resting COX-specific activity and ATP levels in both glycolytic and oxidative skeletal muscle types [188]. Knockout mice had no difference in quadriceps muscle mass, but soleus, a highly aerobic muscle, was significantly smaller. Incremental treadmill exercise tests showed that the wild-type mice were able to run about 38% longer than their *Cox7aH*-knockout counterparts. This was correlated with a 47% decrease in distance and a 47% decrease in workload as compared to that of the wild-type mice. The capillary indices present in the wild-type quadriceps muscle were also found to be significantly higher, in addition to a significant difference in the fiber cross-sectional area and perimeter between the two groups of mice, suggesting that mitochondrial dysfunction in turn causes deterioration of the vascular system feeding them.

Surprisingly, *Cox7aH* was among the most highly upregulated genes in brown fat of mice after cold exposure, but *Cox7aH*-knockout mice exposed to cold were similar in skin temperature, UCP1 production, and other physiological parameters as the controls, demonstrating that nonshivering thermogenesis is not dependent on *Cox7aH* [189]. The pronounced upregulation after cold exposure thus remains puzzling.

**Figure 3:**
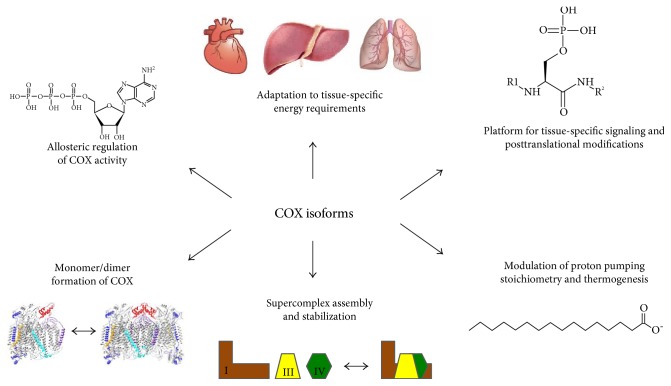
Proposed functions of cytochrome *c* oxidase subunit isoforms. Top left, going clockwise: several COX subunits were proposed to bind to allosteric effector molecules such as ADP and/or ATP, with the most conclusive evidence pointing to COX subunit IV isoforms binding to ADP and ATP at the matrix side, adjusting enzyme activity to energetic demand. COX isoforms also inversely adjust basal enzyme activity to mitochondrial capacity of a tissue, with the activity of COX following the order heart-type < liver-type < lung-type. Due to sequence differences, isoforms may be targeted by signaling molecules such as kinases in a tissue-specific manner. COX subunit VIaL but not VIaH was proposed to bind to the fatty acid palmitate, reducing the stoichiometry of pumped protons per transferred electron, and a similar effect was mediated by VIaH at very high ATP/ADP ratios, which was proposed to contribute to thermogenesis. Finally, COX subunits are key players both in stabilizing the COX dimer (subunits Vb, VIa, and VIb) as seen in the crystal structure of dimeric COX and in supercomplex formation (subunits VIIa, VIIc, and VIII). In both cases, two of the three subunits mediating the contacts have isoforms.

A third isoform of subunit VIIa has been under investigation recently for its potential role in supercomplex formation and regulation. The gene *COX7aR*, also known as *COX7a2L* or *SIG81*, was first identified from a silica-induced gene library [190]. Similar to the ubiquitous liver isoform, COX7AR is expressed in all tissue types, with higher expression levels in those of the kidney and liver. Functional studies remained elusive until recently, when it was proposed that COX7AR was a critical component of supercomplex formation and should be renamed to supercomplex assembly factor I, or SCAFI [191]. Respiratory complexes were screened for proteins that appeared solely in supercomplexes versus free complexes. In the same study, a mutation in the *Cox7aR* gene was discovered in a screen of immortalized mouse fibroblasts resulting in a truncation of the protein from 113 to 111 amino acids, which conferred a defect in supercomplex formation. The authors showed that when *Cox7aR* is silenced or otherwise defective, COX does not participate in supercomplex formation. These results suggest that modulation of COX isoforms may play a critical role in the formation and dispersion of supercomplexes (Figure 3), allowing tight modulation of the electron transport chain through substrate channeling and availability. However, the effect of the truncation remains controversial since two other studies showed that truncated COX VIIaR found in C57BL/6 mice is phenotypically identical to that in nontruncated littermates and as part of supercomplexes [192, 193]. These studies concluded that mice bearing the shortened form of the subunit have normal biogenesis, no related respiratory defects, and normal levels of complex IV-associated supercomplexes, although differences in levels of different supercomplex subtypes were observed. In support of the hypothesis that COX VIIaR is required for supercomplex assembly, however, another recent publication showed that the long form of *COX7AR* was required for interaction of complexes III and IV [194]. Here, it was shown that the individual supercomplexes employ different isoforms to achieve different stoichiometries. Association of complexes III and IV requires *COX7AR*, while complex IV dimers instead utilize COX VIIaL. Another study recently showed that COX VIIaR binds primarily to free complex III and secondarily to COX, where it participates in assembly of the complex III_2_ + IV supercomplex [195]. Recently, the structure of the I + III_2_ + IV supercomplex from pig heart was solved at 4 Å resolution [196], in which COX interacts with both complexes I and III. The position of subunit VIIa appears to be a key in bridging COX with complexes I and III. COX VIIa is close to NDUFB8 of complex I and subunits UQCRC1, UQCRC11, and UQCRB of complex III. However, the resolution of the data set does not allow unambiguous assignment of COX VIIaH or COX VIIa2R (Dr. Yang personal communication), leaving this an open question. There are some discrepancies in the above studies regarding supercomplex composition. Likely, other regulatory mechanisms are in place that contribute to the regulation and stabilization of supercomplexes such as posttranslational modifications, which may explain some of the discrepancies between the above studies. In addition, small but potentially important experimental differences such as precise detergent concentrations and gel running conditions, including temperature and voltage, may affect supercomplex separation and stability.

Finally, a recent study showed that *COX7aR* is a gene that is stress-induced and that its expression also correlates with cancer aggressiveness where it contributes to cancer proliferation and invasion [197]. This suggests that cancer metabolism may be modulated at the level of mitochondrial supercomplexes.

#### 5.2.4. Subunit VIII

The smallest nuclear-encoded subunit is COX VIII (Figure 2), which has three known isoforms in rodents and two in humans. Gene structures and sequence similarities indicate that all three are a result of gene duplications [183]. The primarily expressed liver-type isoform, COX VIIIL (also known as COX VIII2), is expressed ubiquitously in humans [198, 199]. While rodents and most other mammals have a heart-type isoform, COX VIIIH (also known as COX VIII1), with an expression pattern similar to that of COX VIaH and VIIaH in the heart and skeletal muscle, the gene became a pseudogene in the stem of the catarrhines and is thus no longer active in humans [200]. Interestingly, COX VIIIH was also found to be expressed in brown adipose tissue of rats [201].

One important function of COX subunit VIII is to stabilize the supercomplex consisting of complexes I + III_2_ + IV, where it is involved in contacts with subunits NDUFB3, NDUFB7, and NDUFB8 of complex I [196].

The important role of subunit VIII for COX function and stability was further suggested by a recently published clinical study [202]. Here, a female patient with Leigh syndrome-like symptoms who died at age 12 was identified with a homozygous G to C transversion in intron 1 of the ubiquitous *COX8* gene. This mutation disrupts the regular AG acceptor splice site at the end of intron 1, resulting in aberrant splicing, leading to a 49 nucleotide deletion and frameshift of exon 2 and thus a nonfunctional protein. As a consequence, only 10% COX activity was retained in the skeletal muscle and fibroblasts of the patient, which could be restored by expression of wild-type *COX8*.

The presence of a third isoform of COX VIII (also known as COX VIIIC or COX VIII3) was shown in several mammalian species (human, lemur, mouse, and rat) [183]. Phylogenetic analysis based on nucleotide sequence showed high levels of divergence within *COX8-3*, and the protein also had higher amino acid replacement rates compared to the other two isoforms. Of a small number of tissues analyzed to date, *COX8-3* was detected at the highest levels in tissues of the testes followed by those of the pancreas and placenta [202]. Its functional role is currently unknown.

## 6. Conclusions

In this review, we highlight the variation in COX subunit expression in mammals and how tissue-specific and environment-specific conditions necessitate the tight regulation of enzyme activity through differential expression (Figure 3). While decades of research have shaped a thorough understanding of the function and importance of COX, the specific function of many of the nuclear-encoded subunits, as well as their isoforms, remains unclear. Of the 11 nuclear-encoded subunits of COX, six possess tissue- and condition-specific isoforms. We propose that one reason for tissue-specific isoform expression is related to the different capacities of tissues for mitochondria. For example, heart-/skeletal muscle-type COX isoforms of subunits VIa, VIIa, and VIII are expressed in tissues with a high aerobic capacity. Heart and skeletal muscle tissues contain a high density of mitochondria, whereas other tissues including those of the liver and brain, which express the liver-type isoforms, have other specialized functions, which are not compatible with a high mitochondrial load. Because tissues such as those of the liver and brain still fully depend on aerobic energy production, they are equipped with an enzyme containing the liver-type isoforms, which has a higher basal activity [203]. Lung tissue has even fewer mitochondria than that of the liver and expresses the liver-type isoforms of subunits VIa, VIIa, and VIII, together with a lung-specific isoform COX IV-2, leading to yet another increase in basal activity [154]. Therefore, basal activity increases from heart-type over liver-type to lung-type COX, suggesting that one important role of tissue-specific isoforms is to compensate for lack of room for mitochondria in tissues such as those of the liver and lung compared to those of the heart and skeletal muscle. Of the COX isoforms, some additional functional protein data is primarily only available for the isoforms of subunits IV, VIa, VIb, and VIIa—data on the direct enzymatic effects of isoforms of subunits VIII and NDUFA4 has not yet been published. We propose that another functional role of isoforms may be to serve as a platform for tissue-specific signaling and/or allosteric regulation, since amino acid sequence differences between the isoforms may, for example, affect kinase recognition or allosteric effector molecules such as ATP and ADP (Figure 3). In addition, of the three subunits that constitute the primary contact interface with complexes I and III in the supercomplex, that is, VIIaL, VIIc, and VIII, two of these—VIIaL and VIII—are expressed in a tissue-specific manner. This raises the exciting possibility that changes in supercomplex composition, stability, and functionality, including altered metabolic flux, can take place in a tissue-specific manner, adding another layer of regulation to a fundamental bioenergetic process that is most crucial for multicellular organisms. Finally, the emergence of clinical data pointing to individual COX subunits as drivers of critical biological functions and causes of human disease, as well as of individual subunits being potential biomarkers and participants in processes such as oncogenesis, highlights the necessity of continued diligence in their study.

## Figures and Tables

**Table 1:** Nuclear-encoded cytochrome *c* oxidase subunit isoform mutations^1^.

Gene ID	Type of mutation	Disease phenotypes reported
*COX4I2*	Human homozygous missense mutation in 4 patients	Exocrine pancreatic insufficiency; dyserythropoietic anemia; calvarial hyperostosis [163]
*COX4I2*	Mouse homozygous knockout	Reduced airway activity; airway hyporeactivity; lung pathologies [151]
*COX6A1*	5 bp deletion in a splicing element of intron 2 in two consanguineous families	Charcot-Marie-Tooth disease [204]
*COX6B1*	Identical homozygous missense mutation in two patients	Infantile encephalomyopathy [181]
*COX6B1*	Homozygous missense mutation in one patient	Hydrocephalus and cardiomyopathy [182]
*COX7B*	One patient heterozygous for a 1 bp deletion leading to a frameshift in exon 3; one patient heterozygous for a splice site mutation; one patient with a missense mutation in exon 2	X-linked microphthalmia with linear skin lesions [205]
*NDUFA4*	Homozygous splice site mutation in four siblings	Leigh syndrome-like [167]
*COX8*	Homozygous splice site mutation causing frame shift in one patient	Leigh syndrome leukodystrophy and severe epilepsy [202]

^1^Note that additional *heterozygous* mutations have been identified in individual patients with COX deficiency in *COX4I2*, *COX5a*, and *COX6a2* but have not been functionally confirmed as disease causing [164].
